# P085: Surveillance of drug-resistant Salmonella sp and Shigella sp infections in Rwanda

**DOI:** 10.1186/2047-2994-2-S1-P85

**Published:** 2013-06-20

**Authors:** JB Gatabazi

**Affiliations:** 1 Laboratory Department, Rwanda Military Hospital Kigali, Kigali, Rwanda

## Introduction

*Salmonella sp*. and *Shigella sp* infections are public health threat worldwide, particularly in Sub-Saharan Africa including in Rwanda. This study was done to identify *Salmonella sp*. and *Shigella sp*. currently circulating in Rwanda and determine their drug susceptibility pattern.

## Objectives

To determine the prevalence of *Salmonella spp.* and *Shigella spp.* strains circulating in Rwanda and their susceptibilty pattern in Rwanda.

## Methods

196 blood and 24 stool specimens from patients were analyzed in Laboratory of Rwanda for culture isolation, identification and drug-sensitivity testing.

## Results

91 (92.2 %) of them were identified as *Salmonella enterica* serovar Typhi, 10 were *Shigella sp*. The isolates were subsequently subjected to antibiotic susceptibility tests and the strains of *S*.Typhi isolates were found to be susceptible to cefotaxime (100%), and ciprofloxacin (97.9 %) and resistant to nalidixic acid (89.4%), cotrimoxazole (87.2%). With regard to *Shigella* infections, the antibiotics which showed 100% susceptibility to all species identified were ciprofloxacine, cefotaxime, ceftazidine.

## Conclusion

The prevalent *Salmonella* strain circulating in Rwanda is *S*.Typhi and two most useful drugs of choice to treat *Salmonella sp*. and *Shigella sp*. infections in Rwanda are cefotaxime and ciprofloxacin.

## Disclosure of interest

None declared

